# Supplementary material for: Effectiveness of healthcare workers and volunteers training on improving tuberculosis case detection: A systematic review and meta-analysis
Source: PLoS One. 2023 Mar 23;18(3):e0271825. doi: 10.1371/journal.pone.0271825 (PMC10035837; doi:10.1371/journal.pone.0271825)
Supplement: S1 File — (DOCX) [file pone.0271825.s001.docx]

**S1 File: PubMed Advanced Search Terms**

(((((((((((((("tuberculosis"[MeSH Terms] OR "tuberculosis"[All Fields]) AND "case"[All Fields] AND detection[All Fields]) OR (("tuberculosis"[MeSH Terms] OR "tuberculosis"[All Fields]) AND "case"[All Fields] AND notification[All Fields])) OR (("tuberculosis"[MeSH Terms] OR "tuberculosis"[All Fields]) AND ("identification, psychological"[MeSH Terms] OR ("identification"[All Fields] AND "psychological"[All Fields]) OR "psychological identification"[All Fields] OR "identification"[All Fields]))) OR (("tuberculosis"[MeSH Terms] OR "tuberculosis"[All Fields]) AND "case"[All Fields] AND detection[All Fields])) OR (("tuberculosis"[MeSH Terms] OR "tuberculosis"[All Fields]) AND "case"[All Fields] AND detection[All Fields] AND "rate"[All Fields])) OR ("contact tracing"[MeSH Terms] OR ("contact"[All Fields] AND "tracing"[All Fields]) OR "contact tracing"[All Fields])) OR (Active[All Fields] AND "case"[All Fields] AND detection[All Fields])) AND (Home[All Fields] AND ("house calls"[MeSH Terms] OR ("house"[All Fields] AND "calls"[All Fields]) OR "house calls"[All Fields] OR ("home"[All Fields] AND "visiting"[All Fields]) OR "home visiting"[All Fields]))) OR (House[All Fields] AND house[All Fields])) OR (("family characteristics"[MeSH Terms] OR ("family"[All Fields] AND "characteristics"[All Fields]) OR "family characteristics"[All Fields] OR "household"[All Fields]) AND "contact"[All Fields])) AND (TB[All Fields] AND high[All Fields] AND burden[All Fields] AND settings[All Fields])) OR (TB[All Fields] AND high[All Fields] AND burden[All Fields] AND countries[All Fields])) AND (("random allocation"[MeSH Terms] OR ("random"[All Fields] AND "allocation"[All Fields]) OR "random allocation"[All Fields] OR "randomized"[All Fields]) AND ("prevention and control"[Subheading] OR ("prevention"[All Fields] AND "control"[All Fields]) OR "prevention and control"[All Fields] OR "control"[All Fields] OR "control groups"[MeSH Terms] OR ("control"[All Fields] AND "groups"[All Fields]) OR "control groups"[All Fields]) AND ("clinical trials as topic"[MeSH Terms] OR ("clinical"[All Fields] AND "trials"[All Fields] AND "topic"[All Fields]) OR "clinical trials as topic"[All Fields] OR "trial"[All Fields]))) OR "interventions"[All Fields]
