# Supplementary material for: Effectiveness of healthcare workers and volunteers training on improving tuberculosis case detection: A systematic review and meta-analysis
Source: PLoS One. 2023 Mar 23;18(3):e0271825. doi: 10.1371/journal.pone.0271825 (PMC10035837; doi:10.1371/journal.pone.0271825)
Supplement: S1 Table — (DOCX) [file pone.0271825.s002.docx]

**S1 Table: Search key terms**

| Interventions | Tuberculosis case detection | TB high burden settings | Primary healthcare facilities |
| --- | --- | --- | --- |
| - Experimental study - Randomized control trial - Quasi-experiment - Training | - Tuberculosis case notification - Tuberculosis identification - Tuberculosis case detection - Tuberculosis case detection rate - Contact tracing - Active case detection | - Tb prevalent setting - TB high burden countries | - Community - Home to home visiting - House to house - Household contact - Health promotion |
