# Supplementary material for: Effectiveness of healthcare workers and volunteers training on improving tuberculosis case detection: A systematic review and meta-analysis
Source: PLoS One. 2023 Mar 23;18(3):e0271825. doi: 10.1371/journal.pone.0271825 (PMC10035837; doi:10.1371/journal.pone.0271825)
Supplement: S2 Table — (DOCX) [file pone.0271825.s003.docx]

**S2 Table: Data collection and risk assessment form: Intervention review – RCTs and Non-RCTs**

**Article 1**

Authors: Fairal L et al., Year: 2010, Code: RCT-CD01

| S.N | Questions | Yes | No | Unclear | NA | Score |
| --- | --- | --- | --- | --- | --- | --- |
| 1. | Was true randomization used for assignment of participants to treatment groups? | Yes |  |  |  | 1 |
| 2. | Was allocation to treatment groups concealed? | Yes |  |  |  | 1 |
| 3. | Were treatment groups similar at the baseline? | Yes |  |  |  | 0 |
| 4. | Were participants blind to treatment assignment? |  | No |  |  | 0 |
| 5. | Were those delivering treatment blind to treatment assignment? |  | No |  |  | 0 |
| 6. | Were outcomes assessors blind to treatment assignment? |  | No |  |  | 1 |
| 7. | Were treatment groups treated identically other than the intervention of interest? | Yes |  |  |  | 1 |
| 8. | Was follow up complete and if not, were differences between groups in terms of their follow up adequately described and analyzed? | Yes |  |  |  | 1 |
| 9. | Were participants analyzed in the groups to which they were randomized? | Yes |  |  |  | 1 |
| 10. | Were outcomes measured in the same way for treatment groups? | Yes |  |  |  | 1 |
| 11. | Were outcomes measured in a reliable way? | Yes |  |  |  | 1 |
| 12. | Was appropriate statistical analysis used? | Yes |  |  |  | 1 |
| 13. | Was the trial design appropriate? | Yes |  |  |  | 1 |
|  | Total score |  |  |  |  | 10/13 |

**Article 2**

Authors: Ayles H et al., Year: 2013, Code: RCT-CD02

| S.N | Questions | Yes | No | Unclear | NA | score |
| --- | --- | --- | --- | --- | --- | --- |
| 1. | Was true randomization used for assignment of participants to treatment groups? | Yes |  |  |  | 1 |
| 2. | Was allocation to treatment groups concealed? | Yes |  |  |  | 1 |
| 3. | Were treatment groups similar at the baseline? | Yes |  |  |  | 1 |
| 4. | Were participants blind to treatment assignment? | Yes |  |  |  | 1 |
| 5. | Were those delivering treatment blind to treatment assignment? | Yes |  |  |  | 1 |
| 6. | Were outcomes assessors blind to treatment assignment? | Yes |  |  |  | 1 |
| 7. | Were treatment groups treated identically other than the intervention of interest? | Yes |  |  |  | 1 |
| 8. | Was follow up complete and if not, were differences between groups in terms of their follow up adequately described and analyzed? | Yes |  |  |  | 1 |
| 9. | Were participants analyzed in the groups to which they were randomized? | Yes |  |  |  | 1 |
| 10. | Were outcomes measured in the same way for treatment groups? | Yes |  |  |  | 1 |
| 11. | Were outcomes measured in a reliable way? | Yes |  |  |  | 1 |
| 12. | Was appropriate statistical analysis used? | Yes |  |  |  | 1 |
| 13. | Was the trial design appropriate? | Yes |  |  |  | 1 |
|  | Total score |  |  |  |  | 13/13 |

**Article 3**

Authors: Datiko DG and Lindtjørn B, Year: 2009, Code: RCT-CD03

| S.N | Questions | Yes | No | Unclear | NA | Score |
| --- | --- | --- | --- | --- | --- | --- |
| 1. | Was true randomization used for assignment of participants to treatment groups? | Yes |  |  |  | 1 |
| 2. | Was allocation to treatment groups concealed? | Yes |  |  |  | 1 |
| 3. | Were treatment groups similar at the baseline? | Yes |  |  |  | 1 |
| 4. | Were participants blind to treatment assignment? |  | No |  |  | 0 |
| 5. | Were those delivering treatment blind to treatment assignment? |  | No |  |  | 0 |
| 6. | Were outcomes assessors blind to treatment assignment? |  | No |  |  | 0 |
| 7. | Were treatment groups treated identically other than the intervention of interest? | Yes |  |  |  | 1 |
| 8. | Was follow up complete and if not, were differences between groups in terms of their follow up adequately described and analyzed? | Yes |  |  |  | 1 |
| 9. | Were participants analyzed in the groups to which they were randomized? | Yes |  |  |  | 1 |
| 10. | Were outcomes measured in the same way for treatment groups? | Yes |  |  |  | 1 |
| 11. | Were outcomes measured in a reliable way? | Yes |  |  |  | 1 |
| 12. | Was appropriate statistical analysis used? | Yes |  |  |  | 1 |
| 13. | Was the trial design appropriate? | Yes |  |  |  | 1 |
|  | Total score |  |  |  |  | 10/13 |

**Article 4**

Authors: Datiko DG et al., Year: 2017, Code: RCT-CD04

| S.N | Questions | Yes | No | Unclear | NA | Score |
| --- | --- | --- | --- | --- | --- | --- |
| 1. | Was true randomization used for the assignment of participants to treatment groups? | Yes |  |  |  | 1 |
| 2. | Was allocation to treatment groups concealed? | Yes |  |  |  | 1 |
| 3. | Were treatment groups similar at the baseline? | Yes |  |  |  | 1 |
| 4. | Were participants blind to treatment assignment? |  | No |  |  | 0 |
| 5. | Were those delivering treatment blind to treatment assignment? |  | No |  |  | 0 |
| 6. | Were outcomes assessors blind to treatment assignment? |  | No |  |  | 0 |
| 7. | Were treatment groups treated identically other than the intervention of interest? | Yes |  |  |  | 1 |
| 8. | Was follow-up complete and if not, were differences between groups in terms of their follow-up adequately described and analyzed? | Yes |  |  |  | 1 |
| 9. | Were participants analyzed in the groups to which they were randomized? | Yes |  |  |  | 1 |
| 10. | Were outcomes measured in the same way for treatment groups? | Yes |  |  |  | 1 |
| 11. | Were outcomes measured in a reliable way? | Yes |  |  |  | 1 |
| 12. | Was appropriate statistical analysis used? | Yes |  |  |  | 1 |
| 13. | Was the trial design appropriate? | Yes |  |  |  | 1 |
|  | Total score |  |  |  |  | 10/13 |

**Article 5**

Authors: Joshi B et al., Year: 2015, Code: RCT-CD05

| S.N | Questions | Yes | No | Unclear | NA | Score |
| --- | --- | --- | --- | --- | --- | --- |
| 1. | Was true randomization used for the assignment of participants to treatment groups? | Yes |  |  |  | 1 |
| 2. | Was allocation to treatment groups concealed? | Yes |  |  |  | 1 |
| 3. | Were treatment groups similar at the baseline? | Yes |  |  |  | 1 |
| 4. | Were participants blind to treatment assignment? |  | No |  |  | 0 |
| 5. | Were those delivering treatment blind to treatment assignment? |  | No |  |  | 0 |
| 6. | Were outcomes assessors blind to treatment assignment? |  | No |  |  | 0 |
| 7. | Were treatment groups treated identically other than the intervention of interest? | Yes |  |  |  | 1 |
| 8. | Was follow-up complete and if not, were differences between groups in terms of their follow-up adequately described and analyzed? | Yes |  |  |  | 1 |
| 9. | Were participants analyzed in the groups to which they were randomized? | Yes |  |  |  | 1 |
| 10. | Were outcomes measured in the same way for treatment groups? | Yes |  |  |  | 1 |
| 11. | Were outcomes measured in a reliable way? | Yes |  |  |  | 1 |
| 12. | Was appropriate statistical analysis used? | Yes |  |  |  | 1 |
| 13. | Was the trial design appropriate? | Yes |  |  |  | 1 |
|  | Total score |  |  |  |  | 10/13 |

**Article 6**

Authors: Talukder K et., Year: 2012, Code: RCT-CD06

| S.N |  | Yes | No | Unclear | NA | Score |
| --- | --- | --- | --- | --- | --- | --- |
| 1. | Was true randomization used for the assignment of participants to treatment groups? |  | No |  |  | 0 |
| 2. | Was allocation to treatment groups concealed? |  | No |  |  | 0 |
| 3. | Were treatment groups similar at the baseline? | Yes |  |  |  | 1 |
| 4. | Were participants blind to treatment assignment? |  | No |  |  | 0 |
| 5. | Were those delivering treatment blind to treatment assignment? |  | No |  |  | 0 |
| 6. | Were outcomes assessors blind to treatment assignment? |  | No |  |  | 0 |
| 7. | Were treatment groups treated identically other than the intervention of interest? | Yes |  |  |  | 1 |
| 8. | Was follow-up complete and if not, were differences between groups in terms of their follow-up adequately described and analyzed? | Yes |  |  |  | 1 |
| 9 | Were participants analyzed in the groups to which they were randomized? | Yes |  |  |  | 1 |
| 11 | Were outcomes measured in the same way for treatment groups? | Yes |  |  |  | 1 |
| 11 | Were outcomes measured in a reliable way? | Yes |  |  |  | 1 |
| 12. | Was appropriate statistical analysis used? | Yes |  |  |  | 1 |
| 13 | Was the trial design appropriate? | Yes |  |  |  | 1 |
|  | Total score |  |  |  |  | 8/13 |

**Article 7**

Authors: Shargie EB et al., Year: 2012, Code: RCT-CD07

| S.N | Questions | Yes | No | Unclear | NA | Score |
| --- | --- | --- | --- | --- | --- | --- |
| 1. | Was true randomization used for the assignment of participants to treatment groups? |  | No |  |  | 0 |
| 2. | Was allocation to treatment groups concealed? |  | No |  |  | 0 |
| 3. | Were treatment groups similar at the baseline? | Yes |  |  |  | 1 |
| 4. | Were participants blind to treatment assignment? |  | No |  |  | 0 |
| 5. | Were those delivering treatment blind to treatment assignment? |  | No |  |  | 0 |
| 6. | Were outcomes assessors blind to treatment assignment? |  | No |  |  | 0 |
| 7. | Were treatment groups treated identically other than the intervention of interest? | Yes |  |  |  | 1 |
| 8. | Was follow-up complete and if not, were differences between groups in terms of their follow-up adequately described and analyzed? | Yes |  |  |  | 1 |
| 9. | Were participants analyzed in the groups to which they were randomized? | Yes |  |  |  | 1 |
| 10. | Were outcomes measured in the same way for treatment groups? | Yes |  |  |  | 1 |
| 11. | Were outcomes measured in a reliable way? | Yes |  |  |  | 1 |
| 12. | Was appropriate statistical analysis used? | Yes |  |  |  | 1 |
| 13. | Was the trial design appropriate? | Yes |  |  |  | 1 |
|  | Total score |  |  |  |  | 8/13 |

**Article 8**

Authors: Reddy KK et al, Year: 2015, Code: RCT-CD08

| S.N | Questions | Yes | No | Unclear | NA |
| --- | --- | --- | --- | --- | --- |
| 1 | Was true randomization used for assignment of participants to treatment groups? | Yes |  |  |  |
| 2 | Was allocation to treatment groups concealed? | Yes |  |  |  |
| 3 | Were treatment groups similar at the baseline? | No |  |  |  |
| 4 | Were participants blind to treatment assignment? | No | No |  |  |
| 5 | Were those delivering treatment blind to treatment assignment? | No | No |  |  |
| 6 | Were outcomes assessors blind to treatment assignment? | Yes | No |  |  |
| 7 | Were treatment groups treated identically other than the intervention of interest? | Yes |  |  |  |
| 8 | Was follow up complete and if not, were differences between groups in terms of their follow up adequately described and analyzed? | Yes |  |  |  |
| 9 | Were participants analyzed in the groups to which they were randomized? | Yes |  |  |  |
| 10 | Were outcomes measured in the same way for treatment groups? | Yes |  |  |  |
| 11 | Were outcomes measured in a reliable way? | Yes |  |  |  |
| 12 | Was appropriate statistical analysis used? | Yes |  |  |  |
| 13 | Was the trial design appropriate? | Yes |  |  |  |
|  | Total score |  |  |  | 10/13 |

**Article 9**

Authors: Oshi DC et al., Year: 2016, Code: RCT-CD09

| S.N | Questions | Yes | No | Unclear | NA | Score |
| --- | --- | --- | --- | --- | --- | --- |
| 1. | Was true randomization used for the assignment of participants to treatment groups? |  | No |  |  | 0 |
| 2. | Was allocation to treatment groups concealed? |  | No |  |  | 0 |
| 3. | Were treatment groups similar at the baseline? | Yes |  |  |  | 1 |
| 4. | Were participants blind to treatment assignment? |  | No |  |  | 0 |
| 5. | Were those delivering treatment blind to treatment assignment? |  | No |  |  | 0 |
| 6. | Were outcomes assessors blind to treatment assignment? |  | No |  |  | 0 |
| 7. | Were treatment groups treated identically other than the intervention of interest? | Yes |  |  |  | 1 |
| 8. | Was follow-up complete and if not, were differences between groups in terms of their follow-up adequately described and analyzed? | Yes |  |  |  | 1 |
| 9. | Were participants analyzed in the groups to which they were randomized? | Yes |  |  |  | 1 |
| 10. | Were outcomes measured in the same way for treatment groups? | Yes |  |  |  | 1 |
| 11. | Were outcomes measured in a reliable way? | Yes |  |  |  | 1 |
| 12. | Was appropriate statistical analysis used? | Yes |  |  |  | 1 |
| 13. | Was the trial design appropriate? | Yes |  |  |  | 1 |
|  | Total score |  |  |  |  | 8/13 |
