# Supplementary material for: Effectiveness of healthcare workers and volunteers training on improving tuberculosis case detection: A systematic review and meta-analysis
Source: PLoS One. 2023 Mar 23;18(3):e0271825. doi: 10.1371/journal.pone.0271825 (PMC10035837; doi:10.1371/journal.pone.0271825)
Supplement: Attachment — Submitted filename: Response for editor and reviewers.docx [file pone.0271825.s004.docx]

**Rebuttal letter**

We thank the editor and the two reviewers for their comments on our manuscript. The table below is our response to each point raised by the academic editor and reviewers. We hope that we satisfyingly addressed them and that the manuscript will be now suited for publication.

Sincerely,

Desalegne Amare

| S.No | Questions | | Responses |
| --- | --- | --- | --- |
|  | **Editor’s recommendation** | | |
| 1 | Ensure that your manuscript meets PLOS ONE's style requirements, including those for file naming | We have arranged documents according to the PLOS ONE’s style requirement. The name of the files are also arranged based on PLOS ONE’s style for example supplementary files names are corrected as S1 File, S1 Table, S2 Table, Fig 1, Fig2, Fig3 & Fig4 | |
| 2 | Please confirm that you have included all items recommended in the PRISMA checklist | PRISMA checklist was used to include all items. And also we followed PRISMA flow chart. | |
| 3 | Please provide an amended state in your updated Funding Statement | According to your recommendation, funding statement is amended as “Fentie Ambaw Getahun receives support from AMARI (African Mental Health Research Initiative), which is funded through the DELTAS Africa initiative (DEL-15-01). The DELTAS Africa initiative is an independent funding scheme of the African Academia of Sciences (AAS)’s Alliance for accelerating excellence in Science in Africa (AESA) and supported by the New Partnership for Africa’s Development Planning and Coordinating Agency (NEPAD Agency) with funding from the Wellcome Trust (DEL-15-01) and the UK government. The views expressed in this publication are those of the authors and do not necessarily those of the AAS, NEPAD Agency, Wellcome Trust or the UK government.)” | |
| 4 | Funding information should not appear in the Acknowledgments section | The statement that described under acknowledges section is removed because it was misplaced. So the statement is moved to funding session. | |
| 5 | We note that you have stated that you will provide repository information for your data at acceptance. Should your manuscript be accepted for publication, we will hold it until you provide the relevant accession numbers or DOIs necessary to access your data. If you wish to make changes to your Data Availability statement, please describe these changes in your cover letter | There is no published data repository elsewhere. “All data is available in the manuscript”. | |
| 6 | Please amend either the abstract on the online submission form (via Edit Submission) or the abstract in the manuscript so that they are identical | We made identical/the same abstract on the online submission form and in the manuscript. | |
| 7 | Please upload copies of the completed PRISMA checklist as Supporting Information with a file name “PRISMA checklist | We have done that PRISMA checklist as Supporting Information with a file name “PRISMA checklist”. | |

| **Reviewer #1** | | |
| --- | --- | --- |
| 8 | Did you see certain training methods be more successful than others? | In the review process we faced difficulty to determine which training methods are more effective than the other because some of the studies do not show a clear training methods. |
| 9 | What were the training methods used? | - **Fairall LR 2005**: Educational training at health centres and community meetings with messages on childhood TB, using different teaching materials a child TB flip chart with pictures and text messages in the local language. - **Talukder 2012**: Demonstration on Keith Edwards Child TB score chart, administration of the Mantoux test - **Daniel G. Datiko 2009, 2007**: On job training and health education sessions. - **Reddy 2015**: Trained community volunteers but the method of training is not described clearly. - **Oshi DC 2016**: Training was provided to improve the knowledge and skill of health care workers in identifying presumptive childhood TB, but method of training is not clear. - **Shargie 2006**: Group based **interactive lecturing** and **demonstration** and the training focused on; - basic facts about TB: its cause, transmission, symptoms, diagnosis, prevention, treatment, and outcomes - societal perceptions about TB, on how to identify and refer a symptomatic TB suspect, and on how to communicate basic and locally understandable messages about TB - Case-finding, diagnostic procedures, outreach coordination, handling of sputum specimens, interview techniques, and record-keeping. - **Ayles 2013**: The method of training is not clear |
| 10 | Line 25 - Is should be unbold | On line 25 the verb “is” is unbold |
| 11 | Line 27 – professionals | The word professional changed to “professionals” |
| 12 | Line 29 - suggest removing the term RCT as your inclusion criteria also included trials which were not RCTs | Both RCT and Non-RCT are added |
| 13 | Line 56 - second only to COVID19 may not remain true in the long run | Despite Tuberculosis (TB) is a preventable and treatable disease, it remains an important cause of death from an infectious agent, especially, after COVID-19, it is the second deadliest infectious killer. |
| 14 | Line 58 - remove "were" | “Were” is removed |
| 15 | Line 61 - replace "to" with of | Done |
| 16 | Line 66 – diagnosis | Diagnoses changed diagnosis |
| 17 | Line 75 - replace "are investigating" with have investigated | Are investigating changed with “have investigated” |
| 18 | Lines 131-134 - there are grammatical errors here | The authors considered low risk of bias ‘Yes’ either the study protocol is available and all of the studies’ pre-specified outcomes that are of interest in the review have been reported in the pre-specified way or the study protocol is not available but it is clear that the published reports include all of the study’s pre-specified outcomes and all expected outcomes that are of interest in the review. Authors considered high risk of bias ‘No’ if any of the following is fulfilled: Not all of the study’s pre-specified primary outcomes have been reported, one or more primary outcomes is reported using measurements analysis methods or subsets of the data that were not pre-specified, one or more reported primary outcomes were not pre-specified, one or more outcomes of interest in the review are reported incompletely, the study report fails to include results for a key outcome that would be expected to have been reported for such a study. Authors considered unclear if there is insufficient information to permit judgment of ‘Yes’ or ‘No’ |
| 19 | Line 171 – training | “Trainings” changed to “training” |
| 20 | Discussion:  Line 5- workers | The word “works” changed “workers” |
| **Reviewer 2** | | |
| 21 | Very good topic as a lot of health care professionals and volunteers do not focus on TB due to fewer number of cases in the USA when compared to other countries like India | No comments |
| 22 | Statistical analysis was appropriate and presented well. | No comments |
