# Supplementary material for: Effectiveness of healthcare workers and volunteers training on improving tuberculosis case detection: A systematic review and meta-analysis
Source: PLoS One. 2023 Mar 23;18(3):e0271825. doi: 10.1371/journal.pone.0271825 (PMC10035837; doi:10.1371/journal.pone.0271825)
Supplement: Attachment — Submitted filename: Authors response for editor and reviewers(R2).docx [file pone.0271825.s005.docx]

Jun 20, 2022

Editor

PloS ONE

**Subject**: **Submission of revised manuscript**

Thank you for the opportunity to revise our manuscript” Effectiveness of healthcare workers and volunteers training on improving tuberculosis case detection: a systematic review and meta-analysis”. We are grateful for the reviewers’ and editor's comments and believe that the revised manuscript is stronger as a result of their feedback. We are pleased to see that our study is well-recognized by the reviewers as a significant contribution. Below, we have provided point-by-point responses including how and where the text was modified in the main manuscript. Two separate documents, one with track change and the other a clean version are uploaded to the system. We hope that the revised version is now suitable for publication and look forward to hearing from you in due course.

Desalegne Amare

**Point-by-point responses**

**Reviewer #1:** All previously raised comments have been addressed. The data requested should be made available when the manuscript is accepted. But otherwise, no additional revisions are recommended.

**Response**: all relevant documents including the data, quality assessment tools, and risk of bias assessment tools are now included in the main document and supplementary files.

**Reviewer #3:** Present study titled ‘Effectiveness of healthcare workers and volunteers training on improving tuberculosis case detection: a systematic review and meta-analysis is an interesting study focusing on training among healthcare workers. However,

1. Abbreviations and expansion should be mentioned at their first appearance such as WHO.

**Response 1:** We have now spelled out all the abbreviations when they appear for the first time in the manuscript. On page 3 line 60, we have now presented the expanded forms of WHO as ‘World Health Organization (WHO)’

2. Grammatical and punctuation errors such as ‘,’ on page 3 line 60; page 5 line 119.

**Respones2**: We have thoroughly revised the manuscript and edit all the grammatical and punctuation errors. The statements on page 3 lines 60-65 have been now updated as ‘According to the World Health Organization (WHO) report, approximately 10 million people who developed TB in 2020. According to the WHO, 4.1 million people have tuberculosis but have not been diagnosed or have not reported the disease to national authorities. India (41%), Indonesia (14%), the Philippines (12%), and China (8%) were the countries that contributed the most to the global reduction in TB notifications between 2019 and 2020(2)’.

**The stamen on page 5, line 119** has been also now revised as ‘The spreadsheet was checked and refined before data extraction’

3. Repetitive authors list in the bracket on page 4 line 116

**Response3**: authors list is now corrected as (MBS, EWM, TAD, and FAG).

4. Refs 2 and 3 are the same. Please correct the bibliography accordingly.

**Response 4:** The bibliography is now corrected as suggested.

5. Please update references no 3, 4, and 7. Please provide an updated estimate as the WHO TB report 2021 is available.

**Response 5**: The references 3, 4, and 7 are now updated according to the WHO report 2021.

6. Ref 4 and 5 is inaccurate. The correct citation would be ‘Daniel P Chin, Christy L Hanson, Finding the Missing Tuberculosis Patients, The Journal of Infectious Diseases, Volume 216, Issue suppl_7, 1 October 2017, Pages S675–S678’

**Response 6:** We have also checked and revised the citation to the appropriate statements. The statement “As the global community works to end the TB epidemic, the most pressing challenge is that more than 4 million TB patients each year, 40% of all incident cases are not reported to NTPs” is not mentioned by Daniel P Chin and Christy L Hanson as the original source, but these authors used this statement by acknowledging 2016 WHO report. Similarly, 2021 WHO report stated this “4.1 million people have tuberculosis but have not been diagnosed or have not reported the disease to national authorities”. Thus, we used the original sources of information for our citation.

7. On page 3 line 60, 40 % of these cases is not clear. If authors are referring to ‘incidence’ or ‘prevalence’ cases. Please correct it.

**Response 7:** This statement refers to the total number of new TB cases (10 million) worldwide, about 4 million about 40% of the total cases were not reported. Thus, it is not about incidence or prevalence, but it is about the absolute number of new cases missed in the annual report.

8. Refs 4 and 14 are the same. Also, citation seems not appropriate for ref 14 in the introduction and discussion section.

**Response 8**: Thank you it is corrected accordingly.

9. On page 4, line 105, ref 26 is not correct.

**Response 9:** Thank you for this comment again. It is now corrected as “Higgins JP, Thomas J, Chandler J, Cumpston M, Li T, Page MJ, et al. Cochrane handbook for systematic reviews of interventions: John Wiley & Sons; 2019.”

10. List of abbreviations should be complete such as TB is missing in the list

**Response 10**: We have already provided this abbreviation online

11. Fig 1 is not accurate. The screening section should be corrected. Exclusion shell (n =1946) should be after articles screened (n = 2015).

**Response 11**: Thank you for this comment. The flow chart showing the screening process of the articles in now revised and corrected as suggested by the review. Box contains excluded articles after reviewing by using titles and abstracts is placed under-screened articles (2015).

12. Please suggest that Ref 32 Ayles et al (2013) has reported the prevalence of the TB cases, not the TB incidence. Why this has been included in the selected studies. The primary outcome of interest in meta-analysis is the proportion of new cases (on page 5 lines 126-127).

**Response 12**: This study is a randomized control trial that has both intervention and control groups, thus relative risk was calculated manually from the cases and control groups.

13. Please suggest, why RCT and Non-RCT studies were meta-analyzed together. This would have introduced measuring bias in the effect estimates.

**Response 13**: This study includes all interventional studies that might be RCT or NRCT that provided important information for our outcome of interest. However, to avoid bias, we have used strong bias assessment tools.
